# A novel auto‐positioning method in Iodine‐125 seed brachytherapy driven by preoperative planning

**DOI:** 10.1002/acm2.12591

**Published:** 2019-04-24

**Authors:** Xiaodong Ma, Zhiyong Yang, Shan Jiang, Guobin Zhang, Shude Chai

**Affiliations:** ^1^ Centre for Advanced Mechanisms and Robotics, School of Mechanical Engineering Tianjin University Tianjin China; ^2^ Department of Oncology The Second Hospital of Tianjin Medical University Tianjin China

**Keywords:** auto‐positioning, iodine‐125 seed brachytherapy, preoperative planning, treatment planning system

## Abstract

Iodine‐125 seed brachytherapy has great potential in the treatment of malignant tumors. However, the success of this treatment is highly dependent on the ability to accurately position the coplanar template. The aim of this study was to develop an auto‐positioning system for the template with a design focus on efficiency and accuracy. In this study, an auto‐positioning system was presented, which was composed of a treatment planning system (TPS) and a robot‐assisted system. The TPS was developed as a control system for the robot‐assisted system. Then, the robot‐assisted system was driven by the output of the TPS to position the template. Contrast experiments for error validation were carried out in a computed tomography environment to compare with the traditional positioning method (TPM). Animal experiments on Sprague–Dawley rats were also carried out to evaluate the auto‐positioning system. The error validation experiments and animal experiments with this auto‐positioning system were successfully carried out with improved efficiency and accuracy. The error validation experiments achieved a positioning error of 1.04 ± 0.19 mm and a positioning time of 23.15 ± 2.52 min, demonstrating a great improvement compared with the TPM (2.55 ± 0.21 mm and 40.35 ± 2.99 min, respectively). The animal experiments demonstrated that the mean deviation of the seed position was 0.75 mm. The dose‐volume histogram of the preoperative planning showed the same as the postoperative dosimetry validation. A novel auto‐positioning system driven by preoperative planning was established, which exhibited higher efficiency and accuracy compared with the TPM.

## 1 INTRODUCTION

**Figure 1:**
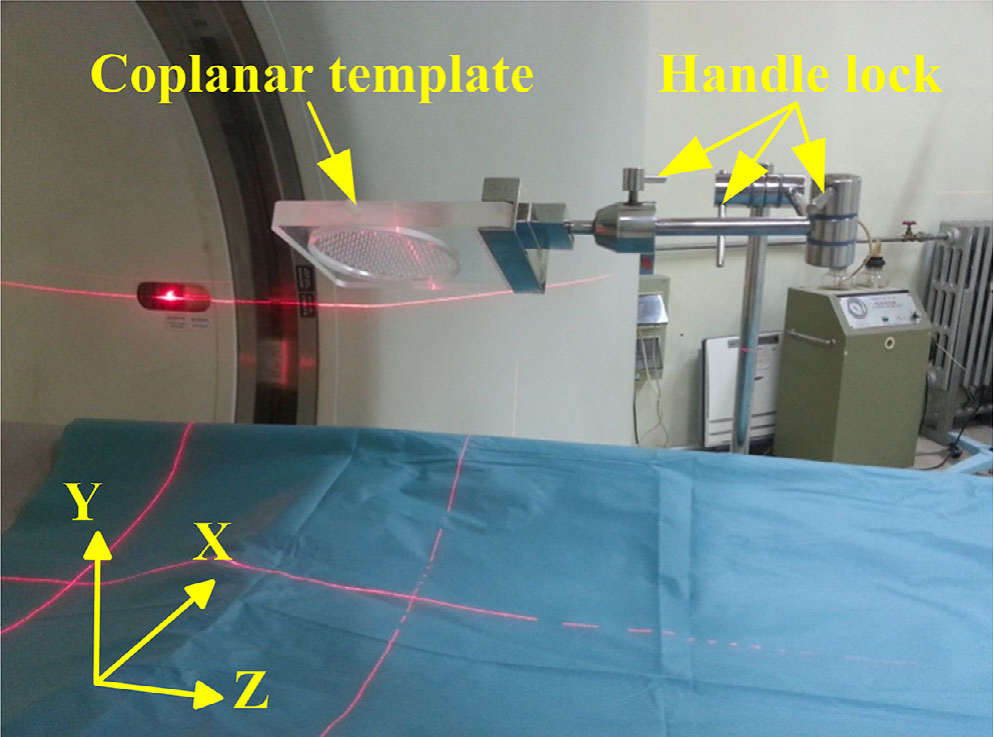
A coplanar template is fixed on a manual mechanism.

Malignant tumors currently represent one of the leading causes of death worldwide and their prevalence is increasing. In total, ~20% of cancer‐associated deaths are caused by lung cancer, and its morbidity has been exhibiting an increasing trend in recent years.^1^ There are various treatment methods, including chemotherapy, external beam radiotherapy, and targeted therapy. Currently, iodine‐125 (^125^I) seed brachytherapy has attracted attention among various methods due to its encouraging clinical efficacy, attributed to its high accuracy and safety.^2^, ^3^, ^4^ Compared with other methods, ^125^I seed brachytherapy can achieve the partial high dose and shorten operative time without affecting the normal tissues.^5^, ^6^, ^7^ In ^125^I seed brachytherapy, a coplanar template (shown in Fig. 1) is used to guide the needle placement. One of the key techniques for successful treatment is the accurate positioning of the template. In addition, high efficiency is also important and worthy of attention.

In the traditional positioning method (TPM), the template is adjusted and fixed using a manual mechanism, as shown in Fig. 1.[8] It is a nonautomatic procedure, and repeated computed tomography (CT) scanning (at least three times) should be performed to ensure that the template has been adjusted to the expected position. The process is completed manually, which is associated with long treatment time and poor accuracy.^9^, ^10^, ^11^

To adjust the template, the initial position and target position of the template must be determined. In TPM, image‐to‐patient registration 12, 13 with the electromagnetic locator (EML) 14, 15, 16 is used to get the initial position and target position of the template. This method involves a treatment planning system (TPS), with three coordinate systems: The role of image‐to‐patient registration is to transform the three coordinate systems into the same system, during which a transformation matrix should be calculated.^17^ In the image‐to‐patient registration, the EML, an external positioning device, is used as an assisting tool. It is widely known that electromagnetic positioning may enable tracking of instruments with high precision. However, there are several drawbacks when used in the CT room. EML is largely affected by ferromagnetic interference sources and the radiology suite.^18^ Therefore, TPM cannot achieve a high accuracy.

Considering the complexity of image‐to‐patient registration using EML, a new positioning system driven by preoperative planning was proposed in this study, referred to as auto‐positioning system driven by preoperative planning (APSDP). A TPS was specifically developed for the new method, in which the preoperative planning could be realized to drive the robot for template positioning. To evaluate the efficiency and accuracy of APSDP, error validation experiments were carried out successfully in a CT environment to compare the new method with TPM. Furthermore, animal experiments were performed for further evaluation.

## 2 MATERIALS AND METHODS

### 2.1 Auto‐positioning system driven by preoperative planning

The APSDP is composed of two different components, a TPS (software) and a robot‐assisted system (hardware). These two components exactly match the surgical process, which may be divided into three parts (pre‐, intra‐, and postoperative). In ^125^I seed implantation treatment, the preoperative planning and the postoperative validation are achieved in the TPS. Subsequently, a robot‐assisted system, developed by our research group, completes the adjustment and fixation of the template during the operation. As mentioned above, accurately positioning the template is critical for successful treatment.

In APSDP, the preoperative planning should be achieved at first using the developed TPS. In TPS, the radiation dose of the ^125^I seeds is calculated according to the “TG43” report recommended by the American Association of Physicists in Medicine.^19^, ^20^ Experiments have been carried out to test the results of dose calculation.^21^ The optimization method for dose distribution is specifically developed for ^125^I seed brachytherapy in thoracoabdominal tumors. The optimization metrics are: at least 90% of the target volume receiving 100% of the prescription dose, at most 50% of the target volume receiving 150% of the prescription dose, and at most 20% of the target volume receiving 200% of the prescription dose. Then, the position of the needles and the target position of the template can be determined. Because the initial position of the template can be easily obtained from CT images, the coordinate systems of the initial position and the target position are transferred to the robot‐assisted system to position the template.

Unlike TPM, there is no image‐to‐patient registration procedure with EML in APSDP. After preoperative planning in TPS, the initial position and the target position are transferred automatically into the same coordinate system with the robot‐assisted system.

**Figure 2:**
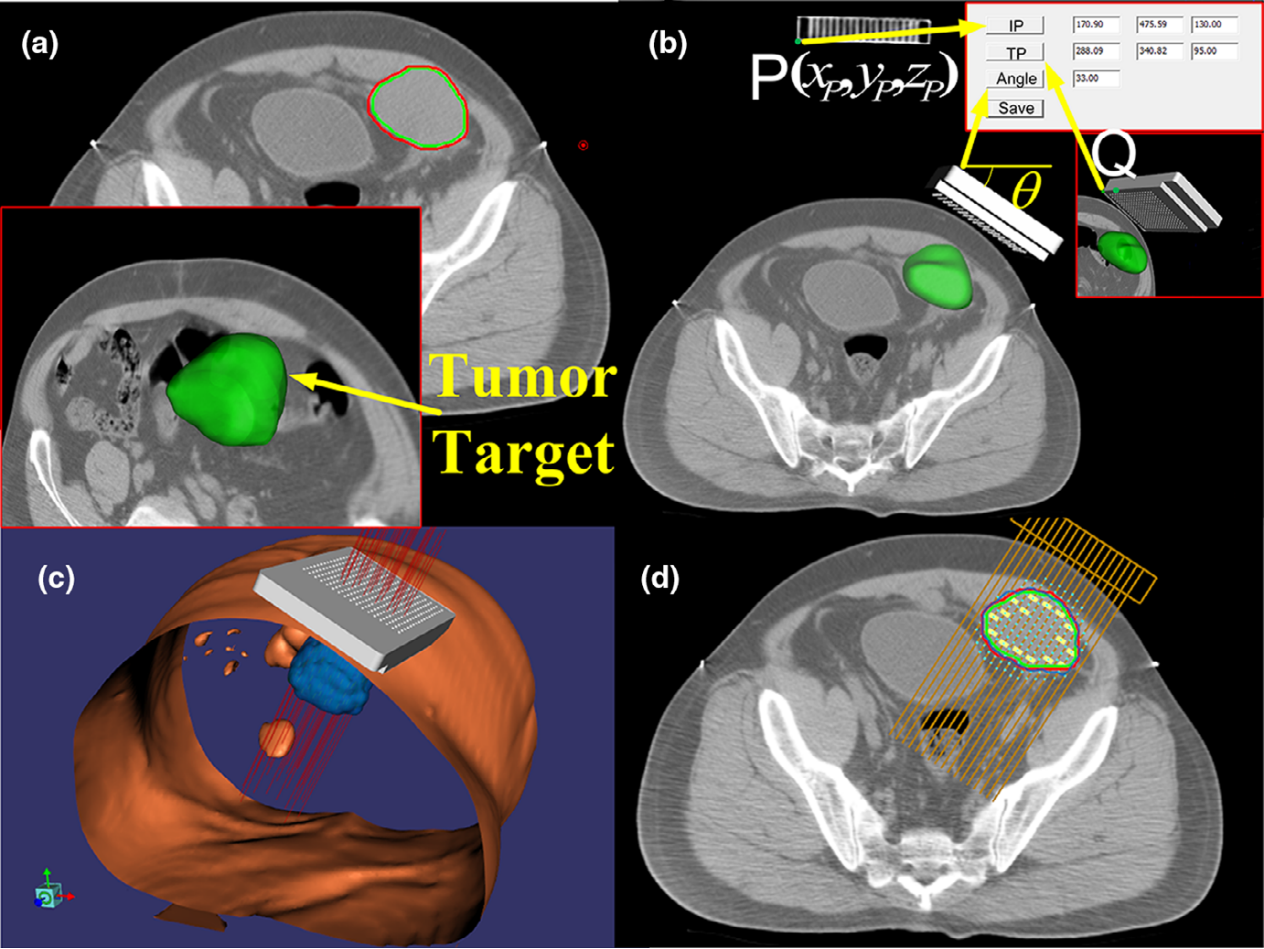
Functions of treatment planning system (TPS). (a) TPS read and displayed the CT images. The tumor target was segmented and reconstructed in the system. (b) In TPS, the initial position and target position were determined. (c) Real‐time display of the template was shown in three‐dimensional space. (d) ^125^I seeds were simulated to be implanted in the tumor along the needle paths and the isodose line (blue line) was displayed to evaluate whether the target was covered by the dose. TPS, treatment planning system; CT, computed tomography; ^125^I, Iodine‐125.

Treatment planning system is the software part of APSDP, which was specifically developed for this method. It was developed in C++ using Visualization Toolkit 22 and Insight Segmentation and Registration Toolkit.^23^ Based on a previous description of APSDP, TPS was developed with several functions to cover the entire procedure of the seed implantation treatment, as shown in Fig. 2. Specifically, there is a navigation module acting as the driving system to realize the connection between the preoperative planning and the robot‐assisted system. The main procedures of the preoperative planning are presented in Figs. 2(b)–2(d). As the patient and the template are scanned in CT together, the template and the needle can be clearly identified in the CT image. In this procedure, the coordinate of the template and the needle is obtained automatically.^24^ The dose planning is crucial before determining the target position of the template, and the ^125^I seeds are then simulated to implant in the CT images as shown in Fig. 2(d). By showing the isodose line, it is easy to determine whether the prescription dose has covered the tumor volume. After adjusting the seed distribution in the CT images to optimal, the target position of the template is also determined.

### 2.2 Workflow of APSDP

**Figure 3:**
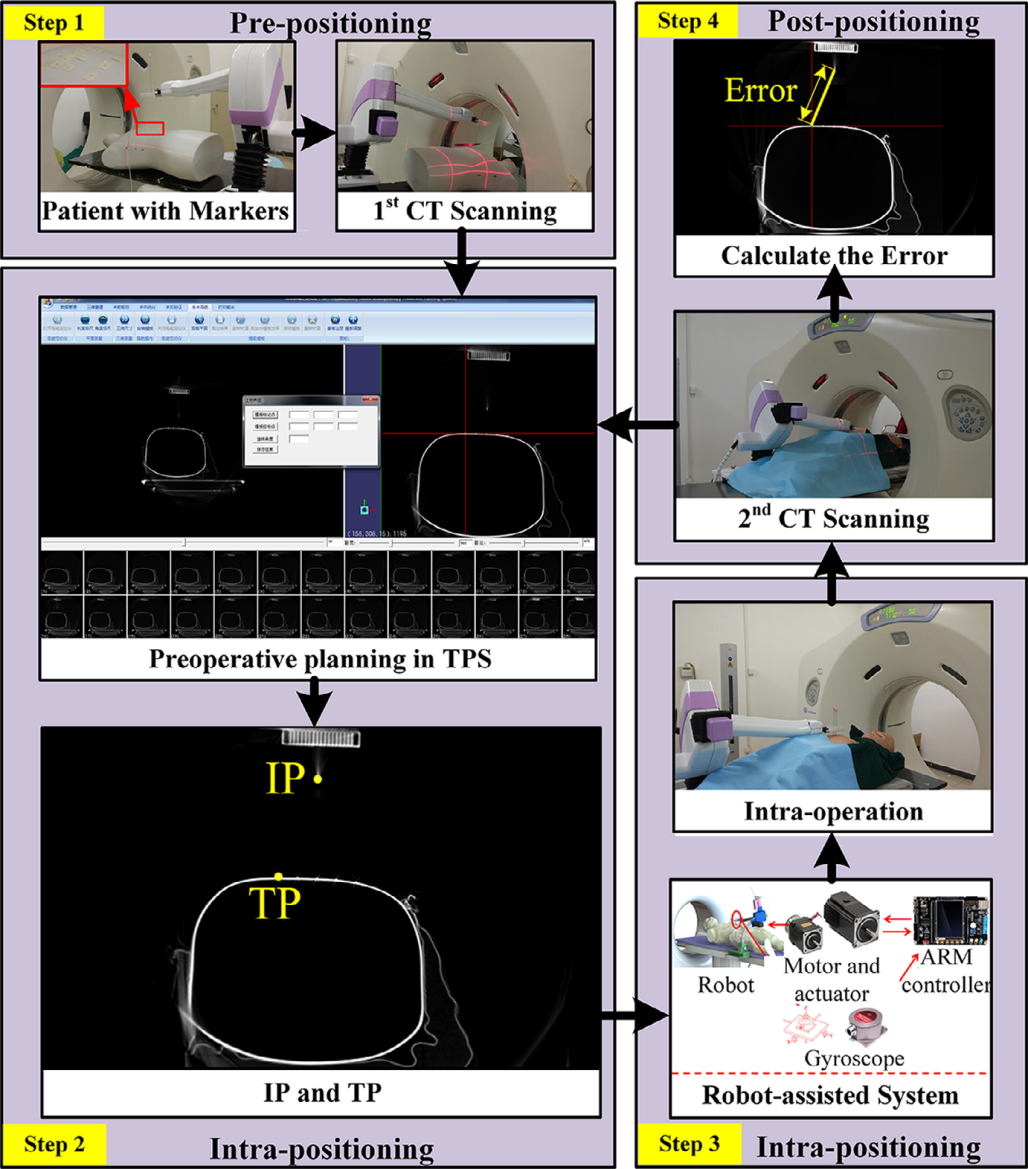
Workflow of APSDP. IP, initial position; TP, target position; APSDP, auto‐positioning system driven by preoperative planning.

**Figure 4:**
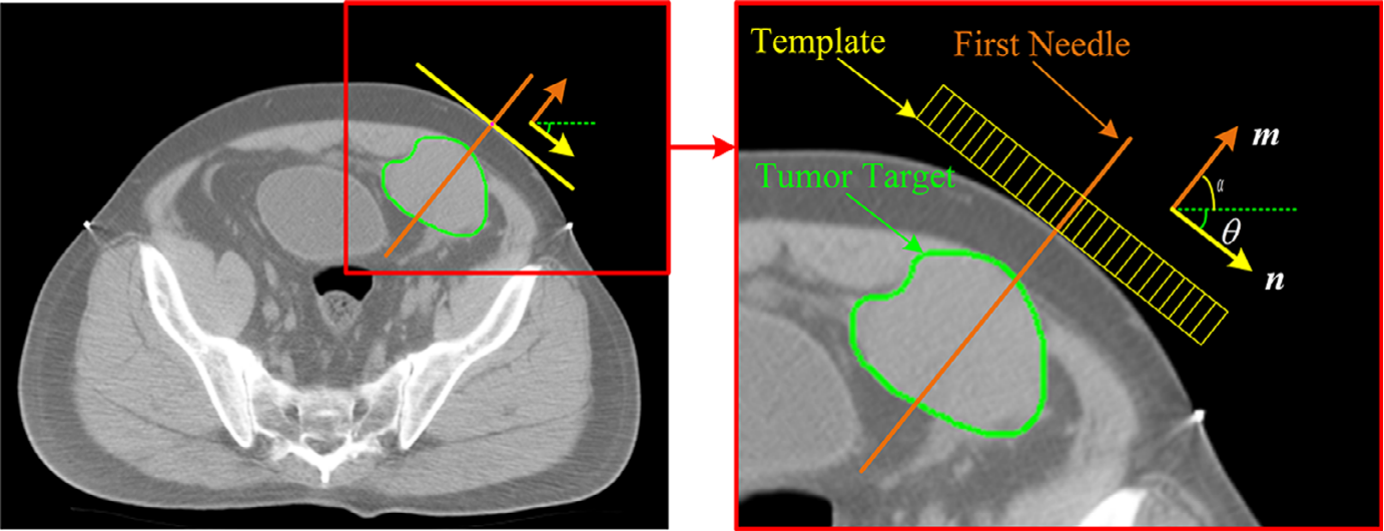
Schematic diagram to determine slant angle. *m* and *n* are the direction vectors of the first needle and the template, respectively.

The workflow of APSDP is shown in Fig. 3 and described as follows:

Step 1: The patient (a human torso dummy was used in this study) and the robot are on the CT table, and a coplanar template is fixed on the end effector of the robot. Then, a CT scanning (first CT scanning) is performed for the patient and the template together.
Step 2: CT images are transferred to the TPS and clinicians perform the preoperative planning. In this step, the target position of the template is determined. The process of obtaining the initial position and target position is shown in Fig. 2(b). The initial position is point P on the template, and its coordinate (*x*_P_ *, y*_P_ *_,_ z*_P_) can be obtained from CT images automatically using the navigation module of the TPS. The target position is point Q on the planned template and its coordinate (*x*_Q_ *, y*_Q_ *_,_ z*_Q_) is determined in preoperative planning. If the template is precisely adjusted to the target position, point P coincides with point Q. The equation to calculate θ is shown in Fig. 4. Before determining the slant angle θ of the template, the position of the first needle (often referred to as the ‘locating’ needle) should be determined based on the widest gap between the ribs, the shortest path of the puncture, and the largest tumor cross‐sectional area. During preoperative planning in TPS, the angle α of a needle may be automatically calculated. Hence, angle θ can be easily calculated.
Step 3: The relative coordinate of the initial position and target position is sent to the robot‐assisted system, and the robot is driven to adjust the template.
Step 4: The first needle is inserted into the tumor target by the clinicians. A CT scan is performed to confirm the position of the needle. Then, additional needles are inserted into the tumor and ^125^I seeds are implanted through these needles. After that, the second CT scanning is performed to confirm the insertion and to make the postoperative validation.

In clinical practice, the first needle is set through the center needle guiding passage of the template to assist the surgeons in judging the accuracy of the template location. Hence, we focused on the slant angle and the position coordinates of the first needle tip but not the template. Because the first needle is perpendicular to the template, and the relative position of the first needle to the template is constant before the template being adjusted to the target position. Specially, in this study, it is assumed that the needle is rigid and does not bend before puncture.

### 2.3 Statistical analysis and error validation

Statistical analysis was performed using *t*‐test in spss software (version: 25.0; Supplier: Microsoft Corporation), with which, it is easy to see whether there is a significant difference between the statistics. *P* < 0.05 was considered to indicate a statistically significant difference.

To examine the accuracy of the robot positioning the template and to ensure the error within an acceptable range (<2 mm), the error validation experiments were performed in the Second Hospital of Tianjin Medical University (Tianjin, China). APSDP was additionally compared with TPM. A human torso dummy was involved in the experiment as mentioned before. In the experiment, for the purpose of controlling a single variable, the manual mechanism (Fig. 1) was replaced with the robot‐assisted system. The error between the planned target position and the real position of the template was used to evaluate the accuracy.

In every test, CT scanning was conducted twice. The purpose of the first CT scan was to determine the initial position and the target position, whereas that of the final scan was to calculate the error [eq. (1)] between the needle tip and the fiducial marker. In this procedure, the coordinates of the needle and the fiducial marker were obtained in the TPS. Then, the slant angle of the needle was easily calculated for comparison with the planned needle angle. Furthermore, the errors in the *X*‐, *Y*‐, and *Z*‐axes were recorded. The errors in the *x*‐ and *y*‐axes were determined by comparing the *X*‐ and *Y*‐coordinates of the needle tip, respectively, with the fiducial marker. The error in the *Z*‐axis was measured using a 3D laser tracker (Leica AT901 LR; Leica Geosystems AG, St. Gallen, Switzerland). As the smallest thickness of the CT images was 1 mm, the coordinate of the needle tip in the *Z*‐axis could not be measured in the CT images (causing a large error of 1 mm).

(1) $$e_{\text{E}}=\sqrt{{\left(x_{\text{P}}-x_{\text{Q}}\right)}^{2}+{\left(y_{\text{P}}-y_{\text{Q}}\right)}^{2}+{\left(z_{\text{P}}-z_{\text{Q}}\right)}^{2}}$$

wherein *e*_E_ is the error between the needle tip and the fiducial marker; (*x*_P_ *, y*_P_ *, z*_P_) the coordinate of the needle tip; (*x*_Q_ *, y*_Q_,* z*_Q_) the coordinate of the fiducial marker, also called the target position.

In TPM, an EML is used to get the coordinate of the initial position and target position. Consequently, through the error validation experiments, the error of APSDP was compared with TPM. If the error of the former was significantly different with the latter, APSDP may be considered sufficiently accurate for clinical application. The time spent on each test was also recorded.

### 2.4 Animal experiments

To further evaluate the performance of APSDP, experiments were also performed using five rats in the Second Hospital of Tianjin Medical University. In the experiments, the ^125^I seed brachytherapy was achieved. The five 6‐month‐old male Sprague–Dawley rats, weighing ~500 g, were obtained from the Tianjin Experimental Animal Center in China. The animals were bred and kept at a controlled temperature of 25°C in a 12‐h light/dark cycle with free access to food and water. For acclimatization, the animals were delivered to the animal facility at least 1 week prior to the study. Approval for this study was obtained from the Peking Union Medical College & Chinese Academy of Medical Science Biomedical Research Ethics Committee (Beijing, China). The rats were anesthetized using intraperitoneal injection of ketamine and xylazine. The ^125^I seeds used in the treatment were produced by Seeds Biological Pharmacy Limited Company (Tianjin, China). The internal dimension of the silver rod was 3.0 × 0.5 mm and the thickness of the titanium capsule was 0.05 mm. The activity of the seed used in these treatments was 2.59 × 10^7 ^Bq or 0.7 mCi. Since the experiments were performed in healthy rats, a part of the liver was selected as the tumor.

**Figure 5:**
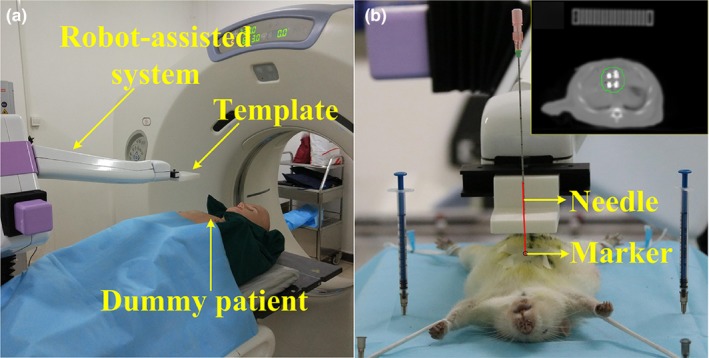
Animal experiments. (a) Platform for animal experiments. (b) The coplanar template was adjusted to the planned position.

The rats were placed in the supine position with all four feet fixed on the platform, as shown in Fig. 5(a). The robot was installed on the CT table, and a coplanar template was fixed on its end effector. The position of the template remained unchanged (at initial position) before being moved to the target position. After the preoperative planning, the robot was driven by the relative coordinate to locate the template. After that, needles were inserted and ^125^I seeds were delivered to the expected position. After CT scanning, postoperative dosimetry validation was achieved. The positions of the seeds were compared with the preoperative planning to calculate the deviation between the two. The dose‐volume histogram (DVH) of preoperative planning was additionally compared with the postoperative DVH. Then, the accuracy of APSDP was easily evaluated.

To analyze the two DVHs, the conformity index (CI) and homogeneity index (HI) were included, which were calculated using eqs (2) and (3), respectively.

(2) $$\text{CI}=\frac{V_{\text{T},\text{ref}}}{V_{\text{T}}}\times \frac{V_{\text{T},\text{ref}}}{V_{\text{ref}}}$$

(3) $$\text{HI}=\frac{D_{2}-D_{98}}{D_{\text{ref}}}\times 100\text{\textbackslash{}\%}\;$$

wherein, *V*_T_ is the total volume of the gross tumor volume (GTV); *V*_ref_ the total volume covered by the prescription dose isodose surface; *V*_T_ *_,_*_ref_ the total volume of the GTV covered by the prescription dose isodose surface; *D*_2_ the dose received by 2% of the GTV, regarded as the maximal dose; *D*_98 _the dose received by 98% of the GTV, regarded as the minimal dose; *D*_ref_ the prescription dose in this plan.

## 3 RESULTS

### 3.1 Error validation experiments

In the error validation experiment, 20 repeated experiments were carried out on a human torso dummy and 20 sets of data were obtained. In APSDP, the errors in *X*‐, *Y*‐, and *Z*‐axis are 0.58 ± 0.14 mm, 0.62 ± 0.16 mm, and 0.58 ± 0.17 mm, respectively. In TPM, the errors in *X*‐, *Y*‐, and *Z*‐axis are 1.38 ± 0.15 mm, 1.53 ± 0.16 mm, and 1.50 ± 0.18 mm, respectively. The angle errors are 0.28°±0.15° and 0.29°±0.16° for APSDP and TPM, respectively. APSDP is better than TPM in terms of the accuracy. The time used for APSDP and TPM are 23.15 ± 2.52 min and 40.35 ± 2.99 min, respectively. In terms of efficiency, APSDP is also better than TPM. The detailed results are summarized in Table 1.

**Table 1:** Results of experiments in two methods (*n* = 20).

Parameter	Unit	APSDP	TPM	*P* a
Angle range	(°)	0–19	0–19	1.000
*X*‐axis error	(mm)	0.58 ± 0.14	1.38 ± 0.15	0.001
*Y*‐axis error	(mm)	0.62 ± 0.16	1.53 ± 0.16	0.001
*Z*‐axis error	(mm)	0.58 ± 0.17	1.50 ± 0.18	0.001
Total error	(mm)	1.04 ± 0.19	2.55 ± 0.21	0.001
Angle error	(°)	0.28 ± 0.15	0.29 ± 0.16	0.844
Time	(min)	23.15 ± 2.52	40.35 ± 2.99	0.001

APSDP, Auto‐positioning system driven by preoperative planning; TPM, traditional positioning method; *n*, number of samples.

a *T*‐test is used for statistical analysis, and *P* < 0.05 means a significant difference.

### 3.2 Animal experiments

The entire procedure of ^125^I seed implantation treatment was achieved successfully in the animal experiments. A total of five rats were used and for each rat, one experiment was carried out. The maximum tumor volume observed was 6.54 cm^3^, and there were no multiple tumors in these experiments. The coplanar template was adjusted to the planned position by the robot [Fig. 5(b)], and the distance between the needle tip and the marker was 0.52 ± 0.07 mm (measured and calculated in the TPS). Subsequently, the clinicians inserted puncture needles according to the preoperative planning. In every test, four needles and six ^125^I seeds were used. The seeds and their distribution in the CT images were clearly identified [Fig. 5(b)]. All seeds were picked up automatically in the TPS, and their coordinates were compared with the preoperative planning. The mean deviation was 0.75 mm, and detailed information is summarized in Table 2. Finally, the postoperative dosimetry validation was achieved after CT scanning. The coverage of the prescription dose to the target is also summarized in Table 2. According to our practice, if at least 90% of the target volume receives 100% of the prescription dose, the target has received a sufficient radiation dose. To evaluate the pre‐ and postoperative DVHs, the comparisons of CI and HI were summarized and analyzed using a *t*‐test, as shown in Table 3.

**Table 2:** The deviation in seed position between preoperative planning and postoperative validation (mm) (*n* = 5).

Rat	*d*_1_	*d*_2_	*d*_3_	*d*_4_	*d*_5_	*d*_6_	Ave_d_	Mean_d_	*D*_100_ (%)
1	0.72	0.71	0.77	0.80	0.53	0.69	0.70 ± 0.09	0.75 ± 0.06	95
2	0.93	0.82	0.78	0.68	0.83	0.64	0.78 ± 0.11		95
3	0.67	0.54	0.91	0.71	0.75	1.37	0.82 ± 0.29		97
4	0.79	0.82	0.86	0.72	0.43	1.01	0.77 ± 0.19		96
5	0.71	0.73	0.39	0.68	0.93	0.60	0.67 ± 0.18		96

*d*, deviation; Ave_d_, Average deviation; Mean_d_, Mean deviation; *D*_100_, the dose received by GTV. *N*, number of samples.

Deviation means the vector sum of the errors in *X*‐, *Y*‐, and *Z*‐axes.

**Table 3:** The comparison of CI and HI (*n* = 5).

Rat	CI		HI		DVH			
	Pre	Post	Pre	Post	Pre/Post (%)			
					*D*_100_	*D*_90_	*V*_100_	*V*_90_
1	0.95	0.94	3.62	3.38	95/93	108/104	95/92	95/93
2	0.97	0.94	3.43	3.25	95/94	108/101	95/92	96/94
3	0.95	0.95	3.48	3.55	97/94	112/103	96/92	96/93
4	0.98	0.97	3.59	3.46	96/94	108/100	95/93	95/93
5	0.95	0.96	3.78	3.42	96/93	111/104	96/94	96/93
Ave	0.960 ± 0.014	0.952 ± 0.013	3.580 ± 0.136	3.412 ± 0.110	96/94	109/102	95/93	96/93
*P* a	0.380		0.230		0.359	0.204	0.435	0.458

Pre: preoperation; Post: postoperation; Ave: average; CI: Conformity Index; HI: Homogeneity Index; *n*: number of samples.

a *T*‐test is used for statistical analysis, and *P* < 0.05 means a significant difference.

## 4 DISCUSSION

The TPM of image‐to‐patient registration is complex and time consuming. Furthermore, EML may be affected by ferromagnetic interference sources and the radiology suite, and thus there is room for improvement. In the present study, APSDP, a novel idea for template positioning in ^125^I seed brachytherapy, is presented, with a focus on efficiency and accuracy. Unlike TPM, the template positioning is driven by the preoperative planning and there is no image‐to‐patient registration procedure with EML. Therefore, the initial position and the target position are obtained through preoperative planning, which is crucial to the whole process.

In APSDP, the developed TPS plays a key role in the whole procedure. First, the complex registration process is replaced by the automatic coordinate transformation in TPS. This design saves significantly more time before the treatment and improves the efficiency. Second, automatically identifying the initial position can prevent errors of manual participation. In the preoperative planning, the reasonable dose planning can ensure an accurate target position of the template. Finally, the selection of functions for the TPS is matched with the whole process of seed implantation treatment. Through accurate template positioning, the clinician is only required to insert the needle according to the preoperative planning. The error validation experiments and the animal experiments have demonstrated the efficiency and accuracy of APSDP.

In the error validation experiments, APSDP (23.15 min; Table 1) saved 17 min compared with TPM (40.35 min; Table 1) in one test. The *t*‐test results demonstrated that these two methods differ significantly in terms of time required (*P* = 0.001, <0.05; Table 1). Therefore, APSDP is more efficient compared with TPM.

The repeated positioning tests demonstrated that the mean error of APSDP (1.04 mm; Table 1) was notably smaller compared with the traditional method (2.55 mm; Table 1). In Table 1, not only the total error but also the error in each axis using APSDP was notably smaller compared with the TPM. Similarly, the results of *t*‐test also demonstrated that that there was a significant difference between the two methods (*P* = 0.001, <0.05; Table 1). The angle errors of the two methods are almost the same (0.28 ± 0.15 and 0.29 ± 0.16 for APSDP and TPM, respectively). Because the rotation angle is different from the position coordinates, it does not require the registration process. Therefore, in both APSDP and TPM, the angle error is the error of the robot itself. To determine whether the angle error of APSDP is better than TMP, the manual mechanism (Fig. 1) should not be replaced with the robot. In the next research, we will try our best to design experiments to finish the verification.

In addition, the mean error of the animal experiments was 0.75 mm (Table 2), which satisfies the clinical requirement (error <2 mm). Comparison between pre‐ and postoperative CI (0.960 vs 0.952; *P* = 0.380, >0.05, respectively; Table 3) and HI (3.580 vs 3.412; *P* = 0.230, >0.05, respectively; Table 3) revealed no significant differences. In addition, the comparison between pre‐ and postoperative DVH (D100: 9579 cGy vs 9399 cGy, *P* = 0.359 > 0.05; D90: 13,341 vs 12,771, *P* = 0.204 > 0.05; V100: 95% vs 93%, *P* = 0.435 > 0.05; V100: 96% vs 93%, *P* = 0.458 > 0.05, respectively; Table 3) demonstrated that there was no significant difference. All these suggested that APSDP is sufficiently accurate to realize preoperative planning. The error validation experiments demonstrated the accuracy of APSDP preoperatively, and the animal experiments demonstrated the accuracy of APSDP intra‐operatively. Consequently, APSDP has better performance to meet the clinical requirements than TPM.

In the clinical surgery, the target volume and the patient body may exhibit different geometric location, shape, and size during the seed implantation. We have also considered this problem. The negative pressure vacuum pad was used to keep the patient’s posture. Before the first CT scanning, the patient lies in the pad, and the pad can make the patient’s posture remain unchanged. This can also reduce changes in tumor shape. At present, we are also researching this issue. If we can avoid the changes in the tumor shape and position or compensate for this change, the accuracy of the method proposed in this paper will be improved. If we make achievements in controlling the tumor location, shape and size, we will report it in the first time.

## 5 CONCLUSION

This study describes an APSDP for ^125^I seed brachytherapy. A TPS was specifically developed to implement this new method. Through the error validation experiments and the animal experiments, APSDP was demonstrated to have higher efficiency and accuracy in ^125^I seed brachytherapy compared with TPM.

## CONFLICT OF INTEREST

All authors declare that they have no conflict of interest.

## ETHICS APPROVAL

Principles of laboratory animal care, as well as specific national laws and regulations, were followed. Approval for this study was obtained from the Peking Union Medical College & Chinese Academy of Medical Science Biomedical Research Ethics Committee (Beijing, China).
